# Some Surgical Contributions

**Published:** 1886-12

**Authors:** Seth N. Jordan

**Affiliations:** Columbus, Ga.


### SOME SURGICAL CONTRIBUTIONS.

BY DR. SETH N. JORDAN, COLUMBUS, GA.

Dieffenbach related once that he was called to see a boy who had received a fracture of the right parietal bone from a fall out of a window to the pavement. He trepanned. The boy recovered, and he believed the boy owed his life to the trepanation. Just one year from the date of the first call, he was again summoned to see the same boy. Examination revealed this time a fracture of the left parietal bone, due to a fall from the original window. This time Dieffenbach did not use the trephine, and he believed that the boy was saved by his conservatism—not trepanning. This is about the position surgeons occupy this day. One author advises, another discourages. Within the past few years it has fallen to my duty to treat a number of fractures of the skull. Some of them have been bored into; the majority not. This month I trephined a boy twelve years old, and as I conceived this case to represent the indications for the operation, I give it. The boy fell from a tree about twenty feet to the ground, receiving a fracture one and a half inches long of the left parietal. I saw him two days after the accident. He then slept all the time; could be roused up, but went off to sleep again. Pulse sixty-five. The skin was not broken. After cutting down to the fracture the bone was elevated, causing an oozing of dark blood. With a chisel the ragged edges were smoothed, spiculæ removed and iodoform sprinkled over the wound. The wound was left open for a while in order that the bleeding could be observed, As it did not bleed and the boy awoke able to converse rationally, the scalp wound was closed and drained with strands of cat-gut. The boy, Marshall Raiford, made a good recovery. It is now pretty generally agreed that an abscess never occurs unless there be an outside wound—a communication with the air. In the case above mentioned, the symptoms of coma were brought about by the pressure of the blood between the dura and the skull. Pressure of the bone could not have produced such profound symptoms. The spiculæ of bone should be removed, just as in any case of a compound fracture. Then closure of blood-vessels and drainage follows again, as in any compound fracture.

A negro boy, Ed. Evans, was struck on the occipital bone with a leaded cane. The day following the accident he became unconscious, slow pulse, breathing deep. I cut down to the bone and found a depressed fracture about the size of a half dollar. The trephine was used. Immediately on removing this, bright blood flowed freely. The bleeding vessel was caught up and a large clot removed. A tampon was placed in the wound. The patient was able to say a few words after the operation, but soon sunk into a comatose state. He died forty-eight hours later. The possibility of an abscess was considered, and a small exploring needle was used, but with only negative result. The *post mortem* examination showed a large clot in the brain itself, going out from the meningea media. The extent of fracture of the internal table can never be judged by that of the external layer. However, it can be pretty accurately relied on to be more extensive.

Another case: Wm. Kimble, of Crawford, Ala, was hit on the head with the handle of an ax. For two days following the blow he was drowsy, but conscious, and complaining only of a headache. A fissure over the left parietal could be felt. He was removed to the city hospital, where on the third day he had a convulsion lasting about five minutes. After this passed off he was in the same condition as before it. Pulse, 80-90. No unfavorable symptoms took place afterward, and he was dismissed in ten days. We know that clots can be re-absorbed, and when not too extensive produce only temporary symptoms. The brain is no longer looked at from afar, but we know that its tissue heals readily without the formation of much new tissue. Fractures of the base of the skull can heal and do just as any other fracture. Subcutaneous fractures of the skull are very frequently complicated with Dupuytren's contusio cerebri, and often heal like other subcutaneous fractures without the formation of pus or any disturbance. Wounds of the head can be placed in identically the same row with other wounds, and demand about the same treatment. Clots, like other foreign bodies, must be removed, arteries must be tied up, oozing must be stopped by means of a tampon, and external antisepsis is forever to be employed. For pyemia, septicemia and meningitis can come in through the air. Iodoform is the antiseptic to be used and it alone. Use iodoform gauze in the place of sponges whenever possible, and it is possible in very many instances.

The indications for trephining are sharply cut and reduce themselves to this: Apply the trephine in order to make room to remove a clot and tie up bleeding vessels, trephine to empty an abscess and to promote drainage. Loosely trephining, because there chances to be a fracture, is in its decadence.

### NEUROMA OF GREAT SCIATIC NERVE.

Mr. E. P., of this city, has had sciatic pains for the past twelve years, which seemed to radiate in the main from a spot opposite the trochanter. On palpation a tumor was discovered in that region about the size of a hen's egg. Under ether an incision was made down to the tumor, revealing a neuroma lying imbedded in the nerve proper. The tumor was found to be cystic, having about a tablespoonful of fluid contents. It was removed, leaving a continuity of nerve tissue. The patient was walking about the house in one week from operation. Up to the time of the operation the pain had been so acute that sleep was only possible when opium was taken in large doses. Since the operation there is no pain, and for ten days—now one month since the operation—no opium is taken.

### OPERATION ON IMPERFORATE HYMEN.

Mrs. C., married eight years, husband living, was brought to me with the history that something obstructed coitus. On examination the hymen was found to balloon backwards to the portio vaginalis. Imperforate is a misnomer, for there was an opening that permitted the little finger to pass up a half inch. Patient had always menstruated regularly and with little pain. A crucial incision was made and tampons of iodoform cotton packed in and renewed every other day. Patient left for home in one week with a capacious vagina. Dr. J. W. Cameron kindly assisted me and managed the case after the operation.

---

FEMALE DRUG CLERKS IN HOLLAND.—In Holland ladies are gradually usurping the occupations of the pharmaceutical assistants. Out of a total of 55 candidates, 19 out of 31 females and only 8 out of 24 male candidates were successful, in the recent State examination.

